# Withdrawal of infliximab or concomitant immunosuppressant therapy in patients with Crohn's disease on combination therapy (SPARE): a multicentre, open-label, randomised controlled trial

**DOI:** 10.1016/S2468-1253(22)00385-5

**Published:** 2023-01-11

**Authors:** Edouard Louis, Matthieu Resche-Rigon, David Laharie, Jack Satsangi, Nik Ding, Britta Siegmund, Geert D'Haens, Laurence Picon, Peter Bossuyt, Lucine Vuitton, Peter Irving, Stephanie Viennot, Christopher A Lamb, Richard Pollok, Filip Baert, Maria Nachury, Mathurin Fumery, Cyrielle Gilletta, Sven Almer, Shomron Ben-Horin, Yoram Bouhnik, Jean-Frederic Colombel, Erik Hertervig, GETAID and the SPARE-Biocycle research group, Jane Andrews, Miles Sparrow, Rupert Leong, Susan Connor, Graham Radforth-Smith, Peter De Cruz, Jan Preiss, Andrea Stallmach, Thomas Liceni, Olaf Grip, Jonas Halfvarson, Dharmaraj Durai, Fraser Cummings, Christian Seilinger, Miles Parkes, James Lindsay, Guy Lambrecht, Philippe Van Hootegem, Jean-François Rahier, Marie Dewitte, Xavier Hebuterne, Elise Chanteloup, Romain Altwegg, Stephane Nancey, Guillaume Bouguen, Guillaume Pineton de Chambrun, Floriant Poullenot, Xavier Roblin

**Affiliations:** a Department of Gastroenterology, University Hospital CHU of Liège, Liège, Belgium; b Université de Paris, ECSTRRA - CRESS UMR1153, INSERM and SBIM, AP-HP, Hôpital Saint-Louis, Paris, France; c Service d'Hépato-gastroentérologie et oncologie digestive CHU de Bordeaux, Hôpital Haut-Lévêque– Université de Bordeaux, Bordeaux, France; d Translational Gastroenterology Unit, Nuffield Department of Medicine, John Radcliffe Hospital, Oxford, UK; e Department of Gastroenterology, St Vincent's Hospital Melbourne, Melbourne, VIC, Australia; f Department of Medicine, University of Melbourne, Melbourne, VIC, Australia; g Medical Department, Division of Gastroenterology, Infectious Diseases and Rheumatology, Charité - Universitätsmedizin Berlin, Berlin, Germany; h Freie Universität Berlin, Humboldt-Universität zu Berlin, Campus Benjamin Franklin, Berlin, Germany; i Department of Gastroenterology and Hepatology, Amsterdam University Medical Centres, Amsterdam, Netherlands; j Hépato-Gastro-Onco-Entérologie, Hôpital Trousseau, Tours, France; k Imelda GI Clinical Research Center, Imelda General Hospital, Bonheiden, Belgium; l Department of Gastroenterology, Besançon Univeristy Hospital, Besançon, France; m UMR 1098, Franche-Comté University, Besançon, France; n IBD Unit, Department of Gastroenterology, Guy's and St Thomas' NHS Foundation Trust, London, UK; o School of Immunology and Microbial Sciences, King's College London, London, UK; p Department of Gastroenterology, University Hospital of Caen, Caen, France; q Translational & Clinical Research Institute, Newcastle University, Newcastle upon Tyne, UK; r Department of Gastroenterology, Newcastle upon Tyne Hospitals NHS Foundation Trust, Royal Victoria Infirmary, Newcastle upon Tyne, UK; s Gastroenterology, St Georges University Hospital, London, UK; t AZ Delta Hospital, Roeselare, Belgium; u U1286 - INFINITE - Institute for Translational Research in Inflammation, University of Lille, Inserm, CHU Lille, Lille, France; v Department of Gastroenterology, University Hospital of Amiens, Amiens, France; w Peritox, University of Picardie, Amiens, France; x Department of Gastroenterology and Pancreatology, University Hospital of Toulouse Rangueil, Toulouse, France; y IBD-unit, Division of Gastroenterology, Karolinska University hospital, Stockholm, Sweden; z Department of Gastroenterology, Sheba Medical Center, Tel-Aviv University, Israel; aa Department of Gastroenterology, Beaujon Hospital, APHP, Paris Cité University, Clichy, France; ab Department of Gastroenterology, Icahn School of Medicine at Mount Sinai, New York, NY, USA; ac Department of Gastroenterology, Skåne University Hospital, Lund, Sweden

## Abstract

**Background:**

The combination of infliximab and immunosuppressant therapy is a standard management strategy for patients with Crohn's disease. Concerns regarding the implications of long-term combination therapy provided the rationale for a formal clinical trial of treatment de-escalation. Our aim was to compare the relapse rate and the time spent in remission over 2 years between patients continuing combination therapy and those stopping infliximab or immunosuppressant therapy.

**Methods:**

This multicentre, open-label, randomised controlled trial was performed in 64 hospitals in seven countries in Europe and Australia. Adult patients with Crohn's disease in steroid-free clinical remission for more than 6 months, on combination therapy of infliximab and immunosuppressant therapy for at least 8 months were randomly assigned (1:1:1) to either continue combination therapy (combination group), discontinue infliximab (infliximab withdrawal group), or discontinue immunosuppressant therapy (immunosuppressant withdrawal group). Randomisation was stratified according to disease duration before start of first anti-TNF treatment (≤2 or >2 years), failure of immunosuppressant therapy before start of infliximab, and presence of ulcers at baseline endoscopy. The patient number and group of each stratum were assigned by a central online randomisation website. Treatment was optimised or resumed in case of relapse in all groups. Participants, those assessing outcomes, and those analysing the data were not masked to group assignment. The coprimary endpoints were the relapse rate (superiority analysis) and time in remission over 2 years (non-inferiority analysis, non-inferiority margin 35 days). Analyses were done on an intention-to-treat basis. This study is registered with ClinicalTrials.gov, NCT02177071, and with EU Clinical Trials Register, EUDRACT 2014-002311-41. The trial was completed in April, 2021.

**Findings:**

Between Nov 2, 2015, and April 24, 2019, 254 patients were screened. Of these, 211 were randomised and 207 were included in the final analysis (n=67 in the combination group, n=71 in the infliximab withdrawal group, and n=69 in the immunosuppressant withdrawal group). 39 patients had a relapse (eight [12%] of 67 in the combination group, 25 [35%] of 71 in the infliximab withdrawal group, six [9%] of 69 in the immunosuppressant withdrawal group). 2-year relapse rates were 14% (95% CI 4–23) in the combination group, 36% (24–47) in the infliximab withdrawal group, and 10% (2–18) in the immunosuppressant withdrawal group (hazard ratio [HR] 3·45 [95% CI 1·56–7·69], p=0·003, for infliximab withdrawal *vs* combination, and 4·76 [1·92–11·11], p=0·0004, for infliximab withdrawal *vs* immunosuppressant withdrawal). Of 28 patients who had a relapse and were retreated or optimised according to protocol, remission was achieved in 25 patients (one of two in the combination group, 22 of 23 in the infliximab withdrawal group, and two of three in the immunosuppressant withdrawal group). The mean time spent in remission over 2 years was 698 days (95% CI 668–727) in the combination group, 684 days (651–717) in the infliximab withdrawal group, and 706 days (682–730) in the immunosuppressant withdrawal group. The difference in restricted mean survival time in remission was –14 days (95% CI –56 to 27) between the infliximab withdrawal group and the combination group and –22 days (–62 to 16) between the infliximab withdrawal group and the immunosuppressant withdrawal group. The 95% CIs contained the non-inferiority threshold (–35 days). We recorded 31 serious adverse events, in 20 patients, with no difference in frequency between groups. The most frequent serious adverse events were infections (four in the combination group, two in the infliximab withdrawal group, and one in the immunosuppressant withdrawal group) and Crohn's disease exacerbation (three in the combination group, four in the infliximab withdrawal group, and one in the immunosuppressant withdrawal group). No death nor malignancy was recorded.

**Interpretation:**

In patients with Crohn's disease in sustained steroid-free remission under combination therapy with infliximab and immunosuppressant therapy, withdrawal of infliximab should only be considered after careful assessment of risks and benefits for each patient, whereas withdrawal of immunosuppressant therapy could generally represent a preferable strategy when considering treatment de-escalation.

**Funding:**

European Union's Horizon 2020.

## Introduction

Therapeutic strategies for Crohn's disease have evolved over the past decade with mounting evidence that achieving deep remission (defined as clinical, biochemical, and endoscopic remission) is associated with better long-term outcomes.^1^, ^2^, ^3^, ^4^ Combination therapy with infliximab and azathioprine has been shown to be superior to either infliximab or azathioprine monotherapy in achieving clinical remission and endoscopic healing in azathioprine-naive patients, thus supporting the paradigm of early disease management and the use of treatment combinations to increase treatment success.^5^, ^6^, ^7^ However, once remission is achieved, physicians and patients must weigh up the risks and benefits of continuing combination therapy with infliximab and an immunosuppressant (thiopurines or methotrexate).^8^ The risk of opportunistic or serious infections and of lymphoproliferative disorders is of particular concern, with consensus emerging that patients on combination therapy are at greater risk than those on monotherapy.^9^, ^10^ Registry data suggest that de-escalation of drug therapy might reduce the risk of serious drug-related adverse events and might also provide cost savings.^11^, ^12^ By contrast, the risk of disease relapse when discontinuing one or both drugs, or the risk of immunogenicity when discontinuing immunosuppressant therapy and continuing infliximab, is unclear. A randomised trial comparing continuation of combination therapy versus immunosuppressant therapy withdrawal, did not show a higher rate of treatment failure in patients stopping the immunosuppressant therapy.^13^ In the prospective STORI cohort study the risk of relapse was 43·9% at 12 months after infliximab withdrawal in patients treated with combined maintenance therapy.^14^ However, limitations included the absence of a control group and, although infliximab retreatment was allowed, overall time spent in clinical remission over the study period was not assessed. A recent placebo-controlled trial comparing infliximab withdrawal with infliximab continuation in patients in full endoscopic remission confirmed a higher relapse rate in the infliximab withdrawal group (50% *vs* 0).^15^ The role of concomitant immunosuppressant therapy was not specifically examined in that study.^15^

We report the results of a randomised controlled trial comparing the relapse rate and time spent in remission over 2 years in patients with Crohn's disease in clinical remission under combination therapy who were randomly assigned to one of the following groups: continuation of combination therapy of infliximab and immunosuppressant therapy, or withdrawal of infliximab, or withdrawal of immunosuppressant therapy.

**Research in context**

**Evidence before the study**

A treat-to-target strategy and tight disease control have been shown to improve outcomes in Crohn's disease. However, this approach might necessitate long-term use of both biological drugs and immunosuppressant therapy. Combination therapy of anti-TNFα with either thiopurines or methotrexate is well established in this context but is associated with long-term risks and health-care costs. It is not well established whether treatment de-escalation from combination therapy, once sustained steroid-free remission has been achieved, is feasible, nor whether this approach could improve safety and reduce costs without jeopardising disease control. Infliximab withdrawal in patients with Crohn's disease in sustained steroid-free remission has been associated with an increased risk of relapse approximating 50% over 2 years in uncontrolled studies. These studies also provided evidence that remission can be recaptured in most patients by resuming treatment with infliximab, in particular when immunosuppressant therapy had been continued. Other uncontrolled data have concluded that withdrawal of immunosuppressant therapy in patients who had achieved durable remission on combination therapy might not alter relapse rates.

**Added value of the study**

In this randomised controlled study, we confirmed and quantified the increased risk of relapse and loss of time in remission in patients on combination therapy who were randomly assigned to discontinue infliximab, compared with those randomly assigned to continue infliximab in the context of either monotherapy or combination therapy. We demonstrated that immunosuppressant therapy withdrawal was not associated with an increased risk of relapse, nor of drug immunogenicity. We defined risk factors for relapse and for failure of therapy if patients were de-escalated from combination therapy. We also demonstrated that most patients experiencing relapse after infliximab withdrawal rapidly responded to re-treatment and maintained remission over 2 years.

**Implications of all the available evidence**

In patients with Crohn's disease receiving combination infliximab and concomitant immunosuppressant therapy, strategies for treatment de-escalation can be considered in sustained steroid-free clinical remission after assessment of the risk of relapse and potential for effective re-treatment in each individual. Withdrawal of concomitant immunosuppressant therapy does not increase rates of disease relapse or anti-drug antibody formation in those continuing infliximab monotherapy. Withdrawal of infliximab is associated with an increased risk of relapse, but the effects of relapse can be effectively and rapidly treated in most patients. Treatment decisions should be tailored to each patient's circumstances to optimise the balance of potential benefit and harm.

## Methods

### Study design

SPARE was a multicentre, international, open-label, randomised controlled trial involving 64 hospitals in seven countries (Australia, Belgium, France, Germany, Netherlands, UK, Sweden). The aim of the trial was to compare three treatment strategies in patients with Crohn's disease who had achieved steroid-free clinical remission on combination therapy with infliximab and immunosuppressant therapy (thiopurine or methotrexate): continuing combination therapy; stopping infliximab and continuing immunosuppressant therapy as monotherapy; or stopping immunosuppressant therapy and continuing infliximab monotherapy. The study protocol was approved by the relevant ethics committees or institutional review boards of each country and was executed in compliance with the Declaration of Helsinki, Good Clinical Practice guidelines, and applicable local regulations. Protocol deviations (eg, violations of inclusion or exclusion criteria or incorrect treatment or dose) were monitored at study entry and throughout the study. All protocol deviations were assessed in real time for their effect on data integrity and patient safety to determine whether the patient should continue in the study. The study duration was 104 weeks. Major changes in the protocol are explained in the appendix (p 1). Of these, a recalculation of the required sample size was made in November, 2018, following publication of the results of a Crohn's disease patient survey performed in France and in the USA, which provided new information on the acceptability to the patients of time spent with active disease to allow treatment de-escalation, in particular withdrawal of biologics or anti-metabolites, or both.^16^

### Participants

To be eligible, all of the following criteria had to be met at recruitment: an established diagnosis of Crohn's disease, age 18 years or older, treatment with combination therapy with scheduled infliximab (5 mg/kg every 8 weeks over the past 4 months at least) and immunosuppressant therapy (stable dose for the past 3 months) for at least 8 months for luminal Crohn's disease, steroid-free clinical remission for at least 6 months according to retrospective assessment of the patients' medical files, Crohn's Disease Activity Index (CDAI) of less than 150 at baseline,^17^ and use of contraceptive during the whole study for female patients with childbearing potential. Patients gave written informed consent for the study.

Patients were eligible provided that immunosuppressant therapy doses were stable for the past 3 months or more at at least 1 mg/kg for mercaptopurine or 2 mg/kg for azathioprine, or the highest tolerated dose if intolerance to these standard doses; lower doses were also allowed if 6-thioguanine nucleotides (6-TGN) were higher than 235 pmol per 8 × 10^8^ red blood cells. The minimum dose of methotrexate for eligibility was 15 mg per week subcutaneously.

Key exclusion criteria included previous severe acute or delayed infusion reaction to infliximab, perianal fistulae as the main indication for infliximab treatment, active perianal or intra-abdominal fistulae at inclusion defined by active drainage, steroid use within 6 months before screening, and ongoing treatment with steroids, immunosuppressive drugs (other than thiopurines or methotrexate), biologics (other than infliximab), or thalidomide.

### Randomisation and masking

Patients were randomly assigned (1:1:1) to either continue combination therapy (combination group), discontinue infliximab and continue immunosuppressant therapy as monotherapy (infliximab withdrawal group), or discontinue immunosuppressant therapy and continue infliximab therapy as monotherapy (immunosuppressant withdrawal group). Randomisation was stratified according to disease duration before start of the first anti-TNF administration (≤2 years or >2 years), failure of immunosuppressant therapy before the start of infliximab, and presence of ulcers at baseline endoscopy. The patient number and group of each stratum were assigned by a central online randomisation website (Cleanweb). An independent statistician from the Biostatistics Unit of Saint-Louis Hospital (Paris, France) provided computer-generated assignment blocked randomisation lists with the use of a block size of six, unknown to the investigators. Information about allocation was given on the eCRF and confirmed by email. Participants, those assessing outcomes, and those analysing the data were not masked to group assignment.

### Procedures

**Figure 1:**
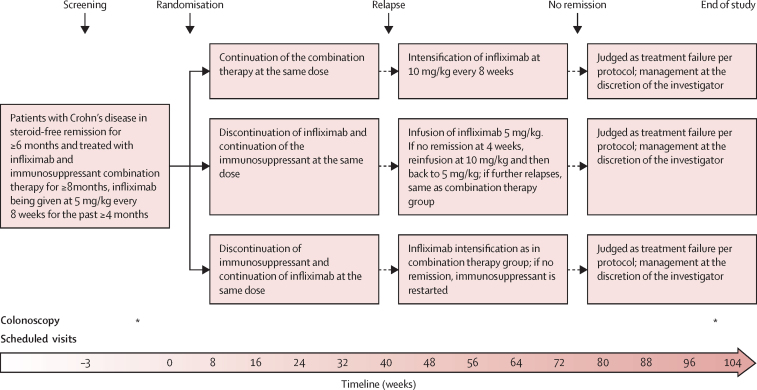
Study design *Indicates that colonoscopy was done at that timepoint.

The study design is shown in figure 1. In the combination group, patients continued scheduled infliximab treatment and immunosuppressant therapy at the same dose as at inclusion. In case of relapse, infliximab dose was increased to 10 mg/kg every 8 weeks. If remission was not achieved 4 weeks after the first 10 mg/kg infliximab infusion (CDAI <150), or if a new relapse occurred after starting the regimen of infliximab 10 mg/kg every 8 weeks, it was classified as treatment failure and the patient was discontinued from the study and treated according to the investigator's discretion. Patients randomly assigned to the infliximab withdrawal group discontinued infliximab and continued immunosuppressant therapy alone at the same dose as at inclusion. In case of relapse, the patient was retreated with an infusion of infliximab 5 mg/kg. If remission was not achieved 4 weeks later (CDAI <150), a second infusion of 10 mg/kg infliximab was administered. If remission was not achieved in an additional 4 weeks, it was considered as a treatment failure, and alternative treatment was instituted according to investigator's discretion. If remission was reached 4 weeks after the first or second reinfusion, infliximab was continued at 5 mg/kg every 8 weeks on a scheduled basis. If a new relapse occurred, the same protocol as in the combination group was applied. In the immunosuppressant withdrawal group, patients discontinued immunosuppressant therapy and continued infliximab alone at the same dose as at inclusion. If a relapse occurred, treatment was intensified as in the combination group. If remission was not recaptured 4 weeks after an infusion of infliximab 10 mg/kg (CDAI <150) or if the patient experienced further relapse under infliximab 10 mg/kg every 8 weeks, immunosuppressant therapy was restarted at the same dose as before inclusion in the trial. If remission was not recaptured 16 weeks later (or earlier in case of disease worsening defined by a further increase of the CDAI of at least 70 points—as compared with the latest relapse visit—or CDAI >300), it was considered as a treatment failure and the treatment was instituted according to investigator's discretion. Infliximab drug levels and anti-drug antibodies (antibodies to infliximab) were determined for secondary endpoints but were not used for treatment optimisation, given the variability in available assays and absence of well-defined thresholds to direct treatment at the time the study was designed.

Study visits were planned every 8 weeks for 104 weeks (end of study visit). The end of study visit took place earlier in case of early study termination. In case of relapse, the end of study visit occurred between week 100 and week 108, at the date closest to week 104. At each visit, patients underwent clinical examination; recording of adverse events; CDAI calculation; quality-of-life assessment by EQ-5D,^18^ Short Health Scale,^19^ and disability index;^20^ and routine blood tests. At baseline and at each study visit, blood samples were obtained for central measurement of high sensitivity C-reactive protein (hsCRP), infliximab levels, and anti-infliximab antibodies, and a stool sample was collected for faecal calprotectin measurement. An ileo-colonoscopy was performed at baseline and end of study visit with assessment by the local investigator of ulcers and measurement of Crohn's Disease Endoscopic Index of Severity (CDEIS),^21^ and Simple Endoscopic Score for Crohn's Disease (SES-CD).^22^ In addition, magnetic resonance enterography (MRE) was also performed at baseline and end of study visit. A pelvic MRI was performed in patients with previous history of perianal fistulising disease.

Infliximab levels and anti-infliximab antibodies were determined by TNF-coated ELISA and by the anti-lambda drug-tolerant ELISA, respectively, as previously described.^23^ All samples were centrally analysed in the Gastro-immunology Laboratory of Sheba Medical Center, Israel. Faecal calprotectin was centrally measured by a particle enhanced turbidimetric immunoassay (Bühlmann fCAL turbo test, Bühlmann, Switzerland); hsCRP was centrally measured by immunoturbidimetric assay (Alinity C, Abbott, USA).

### Outcomes

There were two coprimary endpoints. The first was relapse rate over 2 years, analysed for superiority. Relapse was defined by a CDAI of 250 or higher at any visit or a CDAI between 150 and 250 with an increase of at least 70 points, over two consecutive visits 1 week apart, and a CRP higher than 5 mg/L or a faecal calprotectin higher than 250 μg/g. A new or reopened perianal or entero-cutaneous fistula, an intra-abdominal abscess (size ≥3 cm) or a perianal abscess (size ≥2 cm), and an episode of intestinal obstruction confirmed by imaging and requiring hospital admission were also considered as relapse.

The second coprimary endpoint was the mean restricted time spent in remission during the 104 study weeks, analysed for non-inferiority. For patients who relapsed, this time was computed from the day of randomisation until relapse, and added to the time spent in first and subsequent regained remissions (CDAI <150). Patients failing to recapture remission or fulfilling other definitions of treatment failure terminated the trial and were considered not to be in remission for the remaining weeks of the study (except for the failures linked to treatment side-effects who were censored at the time of failure).

Treatment failure was defined as not achieving remission after treatment escalation for relapse according to the protocol, or any major treatment side-effect leading to treatment cessation, or occurrence of an intra-abdominal abscess (size ≥3 cm) or a perianal abscess (size ≥2 cm), or occurrence of intestinal obstruction requiring surgical resection or endoscopic dilatation.

The main secondary endpoints were: time to relapse from date of randomisation, factors associated with time to relapse, treatment failure rate, time to treatment failure from date of randomisation and factors associated with time to treatment failure, sustained steroid-free remission (defined as CDAI <150 for all trial visits and no steroid use; patients discontinuing the trial for any reason before week 104 were not considered in sustained steroid-free remission), changes between baseline and end of study visit in hsCRP, faecal calprotectin, CDEIS, SES-CD, infliximab trough levels, and anti-infliximab antibodies. The following additional secondary endpoints are not presented in the present manuscript because they are still being analysed: progression of bowel damage, time to relapse according to CRP and calprotectin value, change in the disability score, health-related quality of life, direct and indirect costs, change in blood hsCRP and faecal calprotectin, change in infliximab trough levels and anti-infliximab antibodies, and correlations between a series of biomarkers (proteomics, glycomics, DNA methylation, miRNA, metagenomics), in the three groups of the study.

Adverse events were monitored in all randomised patients during the whole study. Serious adverse events (death, life-threatening hospitalisation and prolongation of hospitalisation, congenital anomaly, spontaneous or elective abortion, significant disability, medical event requiring medical or surgical intervention to prevent serious outcome) were recorded. Adverse events were tabulated by system organ class and preferred term, using the MedDRA dictionary (version 19.0). Incidence and severity of acute or delayed infliximab infusion reactions were assessed according to the Ring and Messmer classification.^24^

### Statistical analysis

The working hypotheses for the two coprimary endpoints were: the relapse rate would be higher in the infliximab withdrawal group as compared with the combination group and the immunosuppressant withdrawal group, but the mean restricted time spent in remission would not be inferior in the infliximab withdrawal group as compared with the combination group and the immunosuppressant withdrawal group. The sample size computation was based on hypotheses around the coprimary endpoints, thus a Bonferroni correction of size 2 was considered to control a global alpha risk of 0·05. We assumed a 2-year relapse rate of 10% in the combination group *vs* 50% in the infliximab withdrawal group, and 20% in the immunosuppressant withdrawal group on the basis of published results.^13^, ^14^, ^25^, ^26^ Given a nominal type I error rate of 0·05 with a statistical power of 0·90, it was computed that 67 patients had to be recruited in each group. Concerning the mean restricted time spent in remission within the first 2 years, a simulation study was conducted under the following assumptions: in the combination group, the first relapse would occur within 2 years in 10% of the patients; the time to relapse would be exponentially distributed (that is, relapse occurs similarly whatever the time spent since randomisation), using the assumed 2-year relapse rate as stated above; 50% of relapses would achieve secondary remission and a second relapse would occur in 50% of these secondary remitters over the rest of the follow-up. Based on these simulated data, the mean time spent in remission restricted at the first 2 years was expected to be 694 days with a standard deviation of 37 days in the combination group. When we designed the trial, the hypothesis was that a threshold of 3% of time over 2 years would be clinically meaningful and that a treatment strategy could be considered as non-inferior below this threshold. According to the results of a patients' survey, performed after the start of the SPARE trial, the majority of the patients (>50%) would accept a difference in restricted mean time spent in remission of 35 days over 2 years (that is, 5% of the mean observed in the combination group).^16^ Hence, in an amendment to the protocol, we chose a 35 days threshold as a maximal margin to demonstrate non-inferiority in mean difference. For this, based on one-sided 97·5% CI, a sample size of 30 patients in each group was necessary, smaller than that calculated for the relapse rate endpoint. Thus, considering 10% of lost to follow up or drop out (not reaching protocol judgement criteria), we planned to enrol 225 patients in the trial.

Continuous variables are summarised as median (IQRs) and categorical variables as counts (%).

Analyses were performed on an intention-to-treat basis. All the patients who had undergone randomisation were analysed in the group they were allocated to. For the primary endpoints, the patients leaving the trial for other reasons than a relapse were censored at the time of drop out, as usually performed in time-to-event analyses. For the secondary endpoint concerning the proportion of patients in steroid-free sustained remission, patients leaving the trial early were considered as not being in sustained steroid-free remission.

Comparisons between groups were performed only to compare the combination group versus the infliximab withdrawal group, and the infliximab withdrawal group versus the immunosuppressant withdrawal group. Failure-free survival and relapse-free survival were estimated by Kaplan–Meier estimators with 95% CIs. Survival curves were tested using log-rank tests. Factors associated with failure-free survival and relapse-free survival were assessed using Cox models. Hazard ratios (HR) with 95% CIs were computed. Multivariable analyses were performed including all factors significantly associated with failure-free survival and relapse-free survival. Mean restricted event times in remission were estimated and compared using the survRM2 R-package.^27^ Differences were computed with 95% CIs using bootstrapping with 1000 replications. For secondary outcomes, differences between groups were assessed using the Wilcoxon rank sum test for quantitative variables and Fisher exact test for qualitative variables. No correction for multiple testing for secondary outcomes was applied. For missing data, multiple imputations were performed using the multiple imputation by chained equation approach. 50 imputed datasets were generated with 20 iterations. Conditional imputations were realised using linear models and predictive mean matching.

All reported p values are two-sided; a p value of less than 0·05 was considered statistically significant. Statistical analyses were performed with SAS (version 9.4) and R (version 4.1.2) statistical software. This study is registered with ClinicalTrials.gov (NCT02177071) and with EU Clinical Trials Register (EUDRACT 2014-002311-41), under the name SPARE.

### Role of the funding source

The funder of the study had no role in study design, data collection, data analysis, data interpretation, or writing of the report.

## Results

**Figure 2:**
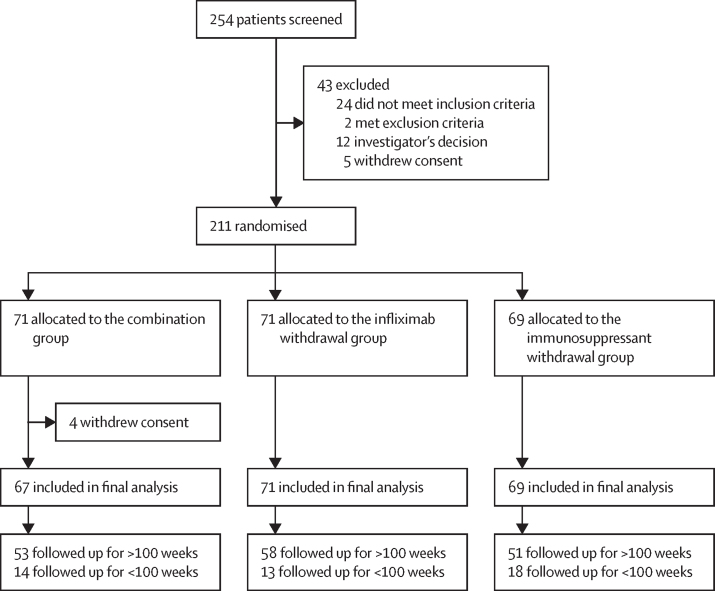
Trial profile Reasons for a follow-up duration shorter than 2 years (<100 weeks, per protocol) in the trial were: Combination group: treatment failure (n=6), patient's decision (n=5), physician's decision (n=2), and last follow-up visit performed too early (n=1); infliximab withdrawal group: treatment failure (n=5), physician decision (n=1), patient's decision (n=4), and last follow-up visit performed too early (n=3); immunosuppressant withdrawal group: treatment failure (n=11), physician's decision (n=1), patient's decision (n=1), last follow-up visit performed too early (n=1), lost to follow-up (n=2), and pregnancy (n=2).

Between Nov 2, 2015, and April 24, 2019, 254 patients were screened, of whom 43 were excluded. 211 patients were randomly assigned either to the combination group (n=71), the infliximab withdrawal group (n=71), or the immunosuppressant withdrawal group (n=69; figure 2). Four patients in the combination group withdrew consent immediately after randomisation and were not included in the analysis. Patient characteristics in the three groups were similar at baseline (table 1). There were 45 patients classified as early terminations within the study. The reasons were: treatment failure as defined per protocol before week 100 and without subsequent follow-up (n=22; six in the combination group, five in the infliximab withdrawal group, and 11 in the immunosuppressant withdrawal group), patient's decision (n=10; five in the combination therapy group, four in the infliximab withdrawal group, and one in the immunosuppressant withdrawal group), physician's decision (n=4; two in the combination therapy group, one in each of the other groups), pregnancy (n=2; both in the immunosuppressant withdrawal group), loss of follow-up (n=2; both in the immunosuppressant withdrawal group); and one visit was missing and the latest visit was performed between 90 and 99 weeks (n=5; one in the combination therapy group, three in the infliximab withdrawal group, and one in the immunosuppressant withdrawal group). There were 11 major protocol deviations. Seven occurred in patients who did not have a relapse: infliximab optimisation to 10 mg/kg every 8 weeks despite absence of confirmed relapse (four in the immunosuppressant withdrawal group and one in the combination group); and restarting infliximab despite absence of confirmed relapse (two in the infliximab withdrawal group). Four occurred in patients who relapsed but were not retreated according to protocol (one in the combination therapy group, two in the infliximab withdrawal group and one in the immunosuppressant withdrawal group).

**Table 1:** Baseline characteristics

		Combination group (n=67)*	Infliximab withdrawal group (n=71)*	Immunosuppressant withdrawal group (n=69)*
Age at randomisation (years)		36 (27–45·5)	32 (25–42·5)	31 (26–44)
Gender				
	Male	37 (55%)	43 (61%)	38 (55%)
	Female	30 (45%)	28 (39%)	31 (45%)
Disease duration (years)		6·4 (3·2–12·7)	6·7 (3·3–10·7)	6·8 (2·9–12·6)
Smoking status				
	Never smoker	36 (54%)	45 (63%)	39 (57%); n=68
	Active smoker	16 (24%)	14 (20%)	17 (25%); n=68
	Previous smoker	15 (22%)	12 (17%)	12 (18%); n=68
Disease location (at the time of infliximab initiation)				
	L1 pure ileal disease (including cecum)	14 (21%)	10 (14%)	13 (19%)
	L2 pure colonic disease	23 (34%)	20 (28%)	21 (30%)
	L3 ileocolonic disease	30 (45%)	41 (58%)	35 (51%)
	L4 upper gastrointestinal tract	9 (13%)	8 (11%)	5 (7%)
Disease behaviour (maximal over disease course)				
	B1 non-stricturing, non-penetrating	42 (63%)	45 (63%)	40 (59%); n=68
	B2 Pure stricturing (without fistula)	10 (15%)	9 (13%)	11 (16%); n=68
	B3 Penetrating (with or without stricture)	15 (22%)	17 (24%)	17 (25%); n=68
Previous perianal disease		12 (18%)	30 (42%)	22 (32%)
Clinically significant gastrointestinal tract stricture at start or during infliximab therapy		8 (12%)	3 (4%)	9 (13%); n=68
Previous intestinal resection		12 (18%)	13 (18%)	17 (25)
Steroid treatment between 12 and 6 months before screening		6 (9%); n=65	1 (1%)	3 (4%)
Failure of the immunosuppressant treatment before the start of infliximab		22 (33%)	23 (32%)	19 (28%)
Immunosuppressant treatments before and during infliximab treatment				
	Azathioprine	54 (81%)	61 (86%)	55 (80%)
	Mercaptopurine	8 (12%)	8 (11%)	8 (12%)
	Methotrexate	5 (7%)	2 (3%)	6 (9%)
Disease duration before the start of the first anti-TNF				
	≤2 years	32 (48%)	38 (54%)	33 (48%)
	>2 years	35 (52%)	33 (46%)	36 (52%)
Duration of infliximab treatment until randomisation (years)		2·3 (1·5–3·6)	2·5 (1·4–4·5)	2·6 (1·6–4·0)
Previous dose increase of infliximab due to loss of response		8 (12%)	6 (8%)	6 (9%)
Loss of response to infliximab in the past 6 months		1 (1%)	0	2 (3%)
Acute or delayed non-severe infusion reaction to infliximab		1 (1%)	2 (3%)	0
Median CDAI		54 (26–79)	38 (18–57·5)	42 (25–83)
Haemoglobin concentration (g/dL)		14 (1·1–14·5)	14·2 (13·7–15·1)	14 (13·3–14·9)
Platelet count (× 10^9^ cells per L)		238 (215·5–299)	255.5 (215·8–299·2)	254 (215–302)
Leucocyte count (× 10^9^ cells per L)		5·5 (4·9–6·7)	5·3 (4·5–6·9)	6·3 (5·3–7·6)
Serum albumin concentration (g/L)		44 (41–46·5); n=59	44 (41–47); n=58	44 (41–46); n=61
Erythrocyte 6-thioguanine concentration (pmol per 8 × 10^8^ red blood cells)		218·5 (122·2–337·2); n=34	295 (259–421); n=41	285 (188–327); n=29
hsCRP (mg/L)		1·24 (0·51–2·8); n=65	1·19 (0·61–2·44); n=68	1·19 (0·52–2·61); n=66
Faecal calprotectin (μg/g)		78·2 (26·1–320·2); n=41	95 (22–289·4); n=47	61·4 (22–195·4); n=53
Infliximab trough level (μg/mL)		3·55 (2·53–5·75); n=66	4·1 (2·5–6·3); n=69	4·1 (2·43–5·73); n=66
Anti-infliximab antibodies (μg/mL)		0·4 (0·3–0·6); n=66	0·5 (0·3–0·7); n=69	0·5 (0·3–0·7); n=66
Presence of ulceration at endoscopy		8 (12%)	8 (11%)	6 (9%)
CDEIS		0 (0–0)	0 (0–0)	0 (0–0); n=68
SES-CD		0 (0–2)	0 (0–1)	0 (0–1)

Data are n (%) or median (IQR). CDAI=Crohn's Disease Activity Index. CDEIS=Crohn's Disease Endoscopic score of Severity. SES-CD=Simplified Endoscopic Score for Crohn's Disease. hsCRP=high-sensitivity C-reactive protein.

* Number of participants is indicated as “; n= ” when not corresponding to n=67 in the combination group, n=71 in the infliximab withdrawal group, and n=69 in the immunosuppressant withdrawal group.

**Figure 3:**
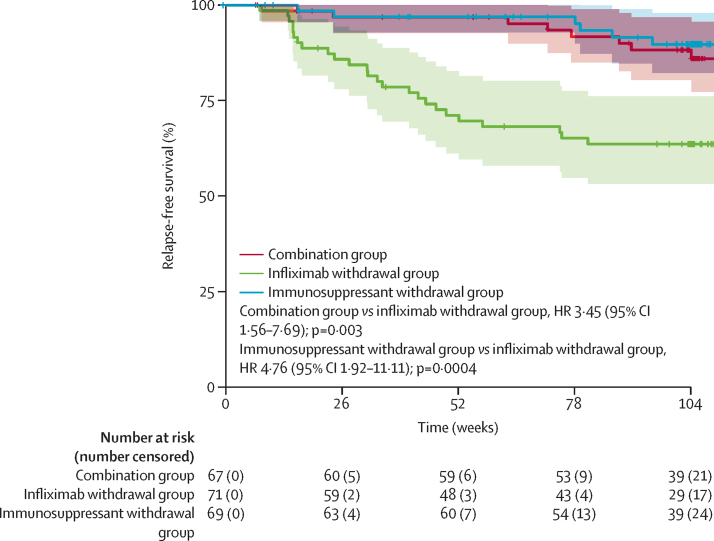
Relapse-free survival Relapse-free survival was estimated by Kaplan–Meier estimators with their 95% CIs in the three groups, over 104 weeks.

39 patients had a relapse: eight (12%) of 67 patients in the combination group, 25 (35%) of 71 patients in the infliximab withdrawal group, six (9%) of 69 patients in the immunosuppressant withdrawal group. 28 patients were retreated or optimised according to protocol. Of these, remission was achieved in one of two patients in the combination group, 22 of 23 patients in the infliximab withdrawal group, and two of three patients in the immunosuppressant withdrawal group. Among the 11 patients who were not retreated, seven were classified as satisfying criteria for treatment failure and four were not retreated according to protocol. Among the patients with a relapse, only one had a second relapse, in the combination group after first treatment optimisation. 2-year relapse rates were 14% (95% CI 4–23) in the combination group, 36% (24–47) in the infliximab withdrawal group, and 10% (2–18) in the immunosuppressant withdrawal group. The corresponding HRs for relapse-free survival were 3·45 (95% CI 1·56–7·69) for the combination group versus the infliximab withdrawal group (p=0·003) and 4·76 (1·92–11·11) for the immunosuppressant withdrawal group versus the infliximab withdrawal group (p=0·0004; figure 3).

**Figure 4:**
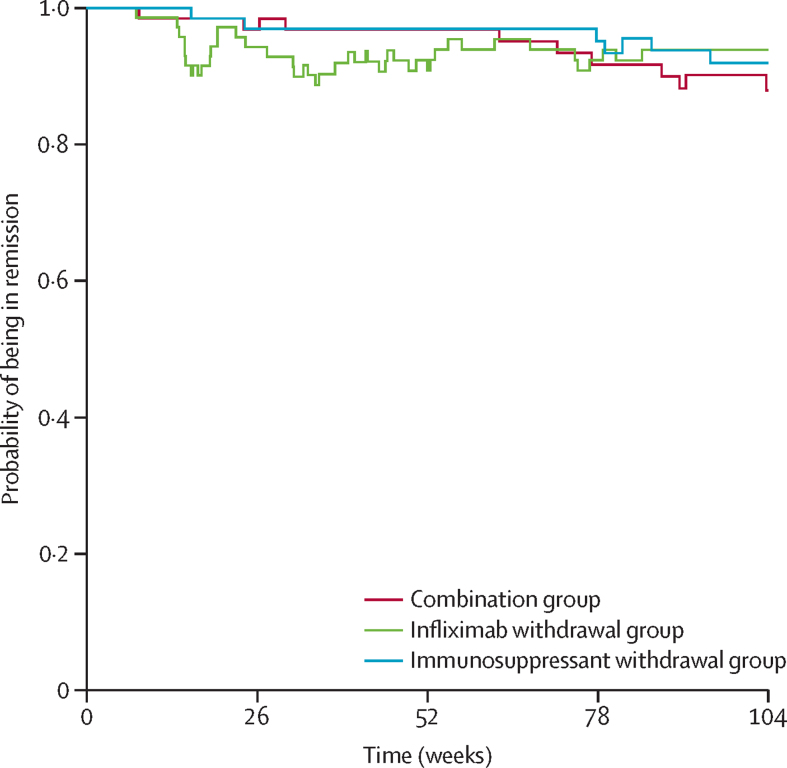
Probability of being in remission by time Patients who had a relapse, were considered not to be in remission until subsequent regained remissions (CDAI <150). Patients for whom remission was not achieved or who met other definitions of treatment failure terminated the trial and were considered not to be in remission for the remainder of the study (except for the treatment failures linked to treatment side-effects who were censored at the time of treatment failure).

The mean time spent in remission was 698 days (95% CI 668–727) in the combination group, 684 days (651–717) in the infliximab withdrawal group, and 706 days (682–730) in the immunosuppressant withdrawal group (figure 4). The difference in terms of restricted mean survival time in remission was –14 days (95% CI –56 to 27) between the infliximab withdrawal group and the combination group and –22 days (–62 to 16) between the infliximab withdrawal group and the immunosuppressant withdrawal group. The 95% CIs both contained the non-inferiority threshold of –35 days.

Factors associated with time to relapse in multivariable analysis were: infliximab withdrawal group (HR 6·67 [95% CI 2·17–20] p=0·001 *vs* the combination group; HR 6·25 [2–20] p=0·002 *vs* the immunosuppressant withdrawal group), young age at diagnosis (<17 years; HR 3·34 [1·43–7·82], p=0·005), hsCRP at baseline as a continuous variable (1·0 mg/L of hsCRP inducing a 0·1 increment of HR; HR 1·10 [1·00–1·20], p=0·039), faecal calprotectin higher than 300 μg/g at baseline (HR 2·62 [1·11–6·18], p=0·028), CDEIS at baseline as a continuous variable (1·0 point of CDEIS inducing a 0·1 increment of HR; HR 1·20 [1·02–1·42], p=0·029).

In patients who discontinued infliximab, only a 6-TGN at baseline higher than 300 pmol per 8 × 10^8^ red blood cells was associated with relapse (HR 0·23 [0·07–0·69]; p=0·009).

**Figure 5:**
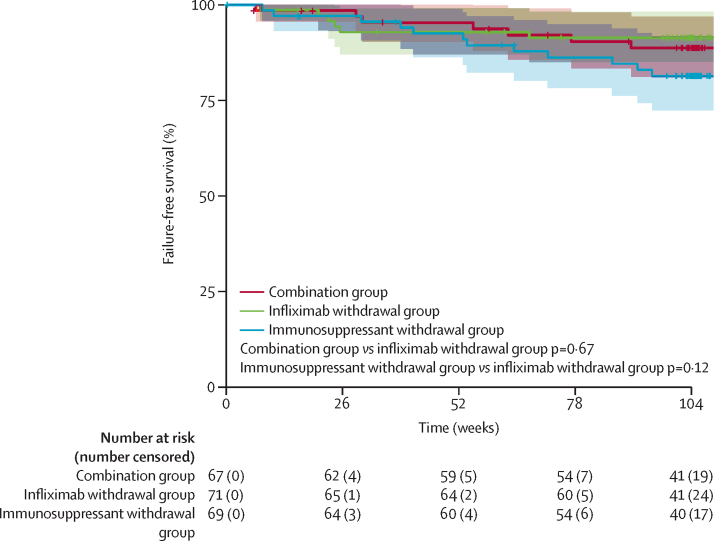
Failure-free survival Failure-free survival was estimated by Kaplan–Meier estimators with their 95% CIs in the three groups of the trial, over 2 years.

Treatment failure was observed in seven (10%) of 67 patients in the combination group (three remissions not recaptured after relapse [including two of them not retreated according to protocol], one obstruction, and three adverse events leading to study drug discontinuation), six (8%) of 71 patients in the infliximab withdrawal group (one remission not recaptured after relapse, two perianal abscesses, one obstruction, two side-effects leading to study drug discontinuation) and in 12 (17%) of 69 patients in the immunosuppressant withdrawal group (one remission not recaptured after relapse, one peri-anal abscess, one obstruction, nine side-effects leading to study drug discontinuation). Figure 5 shows time-to-treatment failure in the three groups.

Factors associated with treatment failure in multivariable analysis were: clinically significant stricture at the time of infliximab induction or during infliximab treatment (HR 3·68 [95% CI 1·41–9·61], p=0·008), and hsCRP at baseline as a continuous variable (1·0 mg/L of hsCRP inducing a 0·1 increment of HR; HR 1·14 [1·08–1·21], p<0·0001). In patients who discontinued infliximab, the only factor associated with failure in multivariable analysis was active smoking (HR 14·28 [1·47–100·00]; p=0·022).

Sustained clinical remission without steroids over 2 years was observed in 36 (54%) of 67 patients in the combination group, 29 (41%) of 71 patients in the infliximab withdrawal group, and 29 (42%) of 69 patients in the immunosuppressant withdrawal group (p=0·17 for the combination group *vs* the infliximab withdrawal group; p=1·0 for the infliximab withdrawal group *vs* the immunosuppressant withdrawal group). Reasons for not achieving sustained clinical remission were steroid use (n=3), CDAI 150 or higher (n=14), and withdrawal before week 100 (n=14) in the combination group; CDAI 150 or higher (n=28), steroid use and CDAI 150 or higher (n=1), and withdrawal before week 100 (n=13) in the infliximab withdrawal group; and steroid use (n=1), CDAI 150 or higher (n=20), steroid use plus CDAI 150 or higher (n=1) and withdrawal before week 100 (n=18) in the immunosuppressant withdrawal group.

Table 2 shows the changes in hsCRP, faecal calprotectin, infliximab trough level, anti-infliximab antibodies, CDEIS, and SES-CD between baseline and the end of study visit for the three study groups. Results including multiple imputations by chained equation, for missing data, are shown in the appendix (p 2). The changes in these parameters between baseline and end of study visit did not differ between the infliximab withdrawal group and the combination group or between the infliximab withdrawal group and the immunosuppressant withdrawal group, except for the infliximab trough levels in the infliximab withdrawal group. Although small, the difference in anti-infliximab antibodies between the infliximab withdrawal group and the immunosuppressant withdrawal group was also statistically significant. In the subgroup of patients in the infliximab withdrawal group who relapsed and were retreated with infliximab (n=23), infliximab trough levels and anti-infliximab antibodies were stable between baseline and end of study visit after infliximab re-institution (median infliximab trough 3·9 μg/mL [IQR 2·5–8·4] at baseline and 4·3 μg/mL [3·6–5·5] at end of study; median anti-infliximab antibodies 0·5 μg/mL [IQR 0·4–0·8] at baseline and 0·4 μg/mL [0·3–0·6] at end of study).

**Table 2:** Difference in endoscopic scores of activity, biomarkers, and drug levels between baseline and end of study visit

		Combination group (n=67)*	Infliximab withdrawal group (n=71)*	Immunosuppressant withdrawal group (n=69)*	**p value (combination *vs* infliximab withdrawal)**	**p value (immunosuppressant withdrawal *vs* infliximab withdrawal)**
hsCRP (mg/L)		..	..	..		
	Baseline	1·24 (0·51 to 2·8); n=65	1·19 (0·61 to 2·44); n=68	1·19 (0·52 to 2·61); n=66	..	..
	End of study	1·41 (0·70 to 4); n=52	1·74 (0·83 to 4); n=58	2 (0·87 to 5·56); n=56	..	..
	Difference (End of study–baseline)	0·05 (−0·48 to 1·61); n=51	0·54 (0·01 to 1·82); n=56	0·6 (−0·18 to 2·3); n=55	0·25	0·51
Faecal calprotectin (μg/g)		..	..	..		
	Baseline	78·2 (26·1 to 320·2); n=41	95 (22 to 289·4); n=47	61·4 (22 to 195·4); n=53	..	..
	End of study	50·7 (22 to 278·7); n=22	148·4 (27·9 to 492); n=30	130·9 (52·4 to 323·3); n=25	..	..
	Difference (End of study–baseline)	0 (−95·8 to 17·9); n=14	1 (−25·9 to 131); n=26	15·3 (−2·7 to 123·8); n=24	0·33	0·47
CDEIS		..	..	..		
	Baseline	0 (0 to 0)	0 (0 to 0)	0 (0 to 0); n=68	..	..
	End of study	0 (0 to 1·9); n=46	0 (0 to 4·8); n=54	0 (0 to 0); n=49	..	..
	Difference (End of study–baseline)	0 (0 to 0·02); n=46	0 (0 to 3·5); n=54	0 (0 to 0); n=49	0·41	0·039
SES-CD		..	..	..		
	Baseline	0 (0 to 2)	0 (0 to 0.5)	0 (0 to 1)	..	..
	End of study	0 (0 to 1); n=65	0 (0 to 4); n=69	0 (0 to 1); n=62	..	..
	Difference (End of study–baseline)	0 (0 to 1); n=65	0 (0 to 2); n=69	0 (0 to 0.8); n=62	0·15	0·29
Infliximab trough (μg/mL)		..	..	..		
	Baseline	3·55 (2·53 to 5·75); n=66	4·1 (2·5 to 6·3); n=69	4·1 (2·43 to 5·73); n=66	..	..
	End of study	3·54 (2·48 to 5·7); n=52	0·07 (0·04 to 3·55); n=54	3·4 (1·8 to 5·8); n=53	..	..
	Difference (End of study–baseline)	0·25 (−1·13 to 2·13); n=52	−3·45 (−5·65 to −0·7); n=53	−0·1 (−2·1 to 1·2); n=53	<0·0001	<0·0001
Anti-infliximab antibodies (μg/mL)		..	..	..		
	Baseline	0·4 (0·3 to 0·6); n=66	0·5 (0·3 to 0·7); n=69	0·5 (0·3 to 0·7); n=66	..	..
	End of study	0·4 (0·28 to 0·6); n=52	0·4 (0·2 to 0·68); n=54	0·5 (0·3 to 0·9); n=53	..	..
	Difference (End of study–baseline)	−0·1 (−0·1 to 0·1); n=52	−0·1 (−0·2 to 0); n=53	0 (−0·1 to 0·2); n=53	0·31	0·012

Data are median (IQR); (n). CDEIS=Crohn's Disease Endoscopic score of Severity. SES-CD=Simplified Endoscopic Score for Crohn's Disease. hsCRP=high-sensitivity C-reactive protein.

* Number of participants is indicated as “; n= ” when not corresponding to n=67 in the combination group, n=71 in the infliximab withdrawal group, and n=69 in the immunosuppressant withdrawal group.

We observed 915 adverse events involving 190 randomised patients. These adverse events had a similar frequency across treatment groups: 282 in patients continuing combination therapy including 92 infections; 338 in patients discontinuing infliximab including 94 infections; and 295 in patients discontinuing immunosuppressants including 73 infections. Two infusion reactions (one grade 1 and one grade 2) were reported in the combination group and two in the immunosuppressant withdrawal group (one grade 1 and 1 grade 2). No infusion reaction was reported in the infliximab withdrawal group.

We observed 31 serious adverse events, which involved 20 patients; these serious adverse events had a similar frequency across the three study groups (table 3). No death nor malignancy was observed. One patient had active tuberculosis in the immunosuppressant withdrawal group and two patients had severe infections (pneumonia and viral pericarditis) in the infliximab withdrawal group.

**Table 3:** Serious adverse events in all randomised patients (n=211)

	**Combination group (n=71)**	**Infliximab withdrawal group (n=71)**	**Immunosuppressant withdrawal group (n=69)**
Infections	4 (6%)*	2 (3%)*	1 (1%)*
Allergic reaction to infliximab	0	0	1 (1%)
Crohn's disease exacerbation	3 (4%)	4 (6%)	1 (1%)
Cancer	0	0	0
Death	0	0	0
Miscellaneous	3 (4%)†	2 (3%)†	10 (14%)†

Data are number of events (%).

* Infections: four cases of appendicitis in the combination group; one case of viral pericarditis and one community-acquired pneumonia in the infliximab withdrawal group; and one report of tuberculosis in the immunosuppressant withdrawal group.

† Miscellaneous includes renal insufficiency (n=2) and gastric sleeve surgery (n=1) in the combination group; ischaemic stroke (n=1) and brachial neuralgia (n=1) in the infliximab withdrawal group; and acute pancreatitis (n=2), alcohol intoxication (n=4), cannabis intoxication (n=1), epilepsy (n=1), sacrococcygeal cyst (n=1), and spontaneous miscarriage (n=1) in the immunosuppressant withdrawal group.

## Discussion

The SPARE trial showed an increased risk of relapse over 2 years in patients with Crohn's disease in clinical remission on combination therapy when discontinuing infliximab as compared with patients continuing infliximab either as monotherapy or in combination with an immunosuppressant therapy. Conversely, discontinuing the immunosuppressant therapy did not affect the relapse rate. Retreatment with infliximab allowed rapid recapture and maintenance of remission in patients who relapsed, and treatment failure rates were similar across treatment groups. Despite this result, the non-inferiority hypothesis for the time spent in remission over 2 years after infliximab withdrawal was rejected. Alongside infliximab withdrawal, other predictors of relapse were young age at onset, and evidence of ongoing inflammation at baseline assessment.

A higher relapse rate after stopping infliximab in patients on combination therapy was expected in light of published data.^14^, ^15^, ^24^ The relapse rate in patients in the infliximab withdrawal group was slightly lower than in the STORI cohort,^14^ and also lower than that reported in a recent placebo-controlled Scandinavian study.^15^ This difference could be explained, at least in part, by the definition of relapse in the present study, which was based not only on CDAI but also on objective inflammatory markers (ie, hsCRP or faecal calprotectin). It is also worth noting that in the Scandinavian study, not all patients were on immunosuppressant therapy when stopping infliximab treatment.^15^

Our working hypothesis upon study design was that, despite this increased risk of relapse, the time spent in remission over 2 years would not be inferior in patients discontinuing infliximab, anticipating a rapid recapture of remission when retreating them and no greater numbers of treatment failure. In patients discontinuing infliximab, retreatment was well tolerated and successful in almost all patients. The treatment failure rate over 2 years was similar and low (around 10%) in the three groups and the difference in the time spent in remission was only around 14 days over 2 years between patients stopping or continuing infliximab. However, the 95% CIs of the differences between study groups in time spent in remission included the non-inferiority threshold, and the non-inferiority hypothesis was thus rejected.

A series of other factors were also associated with relapse in multivariable analysis and should be mentioned: higher hsCRP, faecal calprotectin greater than 300 μg/g, and young age at onset (<17 years). Predictors such as higher CDEIS and SES-CD scores and elevated hsCRP and faecal calprotein levels have been reported in previous studies,^26^ notably in the STORI cohort.^14^ In the group of patients stopping infliximab, only a 6-TGN over 300 pmol per 8 × 10^8^ red blood cells at baseline (which had not been studied in STORI) was a significant negative predictor for relapse, highlighting the key role of immunosuppressant therapy optimisation when withdrawing infliximab. Interestingly, the factors associated with treatment failure were different. They did not include infliximab withdrawal, or faecal calprotectin, but rather active smoking, stricturing behaviour, and elevated hsCRP. In patients randomly assigned to infliximab withdrawal, smoking was the only predictor of failure in the multivariable analysis. If confirmed, these predictors could be important as, in this setting, predicting failure might be more relevant than predicting a simple relapse.

Sustained clinical remission without steroids over 2 years was not significantly different between the three groups. The main reasons for not achieving sustained clinical remission without steroids were early withdrawal and fluctuating CDAI of 150 or higher, which were not systematically associated with a confirmed relapse.

There was no significant difference in change in endoscopic scores of activity, hsCRP, or faecal calprotectin between baseline and end of study visit across groups, suggesting no signal for any subclinical disease escape during these 2 years of treatment de-escalation. Infliximab trough levels at the end of the trial in the retreated patients were very similar to those observed at baseline. The relative stability of infliximab trough levels and low levels of anti-infliximab antibodies in this group might look surprising considering previous concerns regarding the risk of anti-infliximab antibody development and pharmacokinetic loss of response after episodic monotherapy therapy with infliximab.^28^, ^29^ However, patients who discontinued infliximab were in long-term remission and maintained on immunosuppressant therapy after infliximab withdrawal, which are two parameters that have been previously associated with a low risk of immunogenicity.^7^, ^14^, ^30^

The lack of a difference in relapse rate (and in changes between baseline and end of study in endoscopic scores, markers of inflammation, infliximab trough levels, and anti-infliximab antibodies) between patients continuing and those stopping immunosuppressant therapy when being on infliximab confirms the lack of clinical benefit of continuing immunosuppressant therapy in patients treated with infliximab for a median of more than 2 years. The clinical outcome is in agreement with a previous controlled study,^13^ but the data for changes in inflammatory markers and infliximab trough levels differs,^13^ and we point out that these results might not be extrapolatable to all patients treated with combination therapy of infliximab and immunosuppressant therapy for shorter periods of time.

Our study was not powered to identify differences in safety profiles accurately across the treatment groups, but no new safety signals were observed in any group. Interestingly, no acute infusion reaction was observed in the group of patients discontinuing infliximab and being retreated after a drug holiday period. This observation is consistent with the absence of development of anti-infliximab antibodies in that group and also with previous findings.^14^

We acknowledge that our trial was limited by the absence of blinding. This limitation might have impacted the assessment of the primary endpoint based on CDAI variations. Indeed, subjective measures are included in CDAI, which could have been influenced by the knowledge of the administered treatment. Subjectivity of the endpoint was limited by the mandatory confirmation of relapse with one objective marker of inflammation (hsCRP or faecal calprotectin). Another limitation is the relatively low number of events, reducing accuracy in determining risk factors associated with either relapse or treatment failure. We also acknowledge some missing data for biomarkers, particularly faecal calprotectin at baseline and end of study, and endoscopy at end of study. Finally, although infliximab trough levels and anti-infliximab antibodies were measured for secondary endpoints in the trial, they were not used for treatment optimisation.

In conclusion, the results of the SPARE trial show that discontinuation of infliximab in patients on combination therapy is associated with an increase in the risk of relapse compared with patients continuing combination therapy and also with those switching to infliximab monotherapy. We were able to identify clinical characteristics of patients with high risk of relapse and failure. In the group of patients experiencing relapse, the large majority responded promptly to infliximab retreatment, so that the loss of time spent in remission was only 2–3 weeks over 2 years. These results provide information to help guide treatment decisions for clinicians and for patients on sustained remission under combination therapy with infliximab and immunosuppressant therapy. An informed decision on treatment de-escalation by withdrawing either infliximab or immunosuppressant therapy might be agreed, taking into account these findings together with patient preferences, clinical characteristics, and other factors including national health policy guidelines and economic considerations.

For the **Cleanweb** see https://cleanweb.tentelemed.com/Ctms-cleanweb/portal/login?page_target=com.tentelemed.portal.pages.JumpBoardPage

## Data sharing

Collected data (deidentified patients' data, with dictionary defining each field in the set, in an Excel extracted database) will be made available to others for meta-analyses or complementary analyses, with publication, after approval of a proposal, with a signed data access agreement. The study protocol, statistical analysis plan, and informed consent form template will also made available upon request to the corresponding author.

## Declaration of interests

EL has received educational and research grants from Janssen, Pfizer, AbbVie, and Takeda, Fresenius-Kabi; speaker fees from AbbVie, Dr Falk Pharma, Ferring, Janssen, Pfizer, Celgene, Bristol Myers Squibb (BMS), Galapagos, and Takeda; advisory board fees for AbbVie, Celgene, Ferring, Janssen, BMS, Pfizer, Takeda, Gilead-Galapagos, Arena, and Elli Lilly; and consultancy fees from AbbVie. JS reports research support from European Commission Horizon 2020 programme. ND has received research grants from AbbVie, Janssen, and Takeda; speaker fees from AbbVie, Celltrion, Pfizer, Janssen, and Takeda; and advisory board fees from AbbVie, Dr Falk Pharma, and Janssen. BS has served as a consultant for AbbVie, Arena, BMS, Boehringer, CT Scout, Galapagos, Gilead, Janssen, Lilly, and PredictImmune; and has received speaker's fees from AbbVie, CED Service GmbH, Dr Falk Pharma, Ferring, Janssen, Materia Prima, Pfizer, and Lilly. GD'H reports consultancy activities for AbbVie, Agomab, Alimentiv, AstraZeneca, AM Pharma, AMT, Arena Pharmaceuticals, BMS, Boehringer Ingelheim, Celltrion, Eli Lilly, Exeliom Biosciences, Exo Biologics, Galapagos, Index Pharmaceuticals, Kaleido, Roche, Gilead, GlaxoSmithKline, Gossamerbio, Pfizer, Immunic, Johnson & Johnson, Origo, Polpharma, Procise Diagnostics, Prometheus laboratories, Prometheus Biosciences, Progenity, and Protagonist; speaker's bureau for AbbVie, Arena, Galapagos, Gilead, Pfizer, BMS, and Takeda; and fees for data monitoring board activities for Galapagos, AbbVie, Astrazeneca, and Seres Health. PB has received financial support for research from AbbVie, Amgen, Celltrion, Mylan, Pfizer and Takeda; lecture fees from AbbVie, Celltrion, Galapagos, Janssen, Lilly, Pentax, and Takeda; advisory board fees from AbbVie, Arena Pharmaceuticals, BMS, Celltrion, Dr Falk Pharma, Galapagos, Janssen, Lilly, Pentax, PSI-CRO, Roche, Takeda, and Tetrameros. LV has received fees from AbbVie, Amgen, Biogen, Mylan, Takeda, MSD, Janssen, Pfizer, Ferring, and Galapagos. PI has received honoraria for talking on behalf of AbbVie, BMS, Celgene, Celltrion, Dr Falk Pharma, Ferring, Galapagos, Gilead, MSD, Janssen, Lilly, Pfizer, Takeda, Tillotts, Sapphire Medical, Sandoz, Shire, and Warner Chilcott; and for acting in an advisory capacity to AbbVie, Arena, Boehringer-Ingelheim, BMS, Celgene, Celltrion, Connect Biopharma, Genentech, Gilead, Hospira, Janssen, Lilly, MSD, Pfizer, Pharmacosmos, Prometheus, Roche, Sandoz, Samsung Bioepis, Takeda, Topivert, VH2, Vifor Pharma, and Warner Chilcott. SV has received consulting or lecture fees for AbbVie, Amgen, Sandoz, Janssen, MSD, Pfizer, Celltrion, and Takeda. CAL has received grants from Genentech, AbbVie, Eli Lilly, Pfizer, Roche, UCB Biopharma, Sanofi Aventis, Biogen IDEC, Orion OYJ, and AstraZeneca; grants and personal fees from Janssen, Takeda, and Ferring; and personal fees from Dr Falk Pharma, outside the submitted work. FB has received grant or research support from AbbVie, Amgen, Janssen, and Takeda; honoraria from AbbVie, Dr Falk Pharma, Arena, Celgene, Mundipharma, Ferring, Vifor, Janssen, Merck Sharp & Dohme, Pfizer, Norgine, and Takeda. MN received board membership, consultancy, or lecture fees from AbbVie, Amgen, Arena, Biogen, CTMA, Celltrion, Ferring, Fresenius, Janssen, Mayoli-Spindler, MSD, Pfizer, Sandoz, and Takeda. MF received consulting or lecture fees for AbbVie, Amgen, Biogen, Gilead, Sandoz, Janssen, MSD, Pfizer, Ferring, Lilly, Tillots, Celltrion, Fresenius, Galapagos, and Takeda. CG received consulting or lecture fees from AbbVie, Amgen, Celltrion, Biogen, Fresenius, Gilead, Janssen, MSD, Mylan, Pfizer, Takeda, and Vifor. SB-H has received advisory board or consulting fees from AbbVie, Takeda, Janssen, Celltrion, Pfizer, GlaxoSmithKline, Ferring, Novartis, Roche, Gilead, NeoPharm, Predicta Med, Galmed, Medial Earlysign, and Eli Lilly, and research support from AbbVie, Takeda, Janssen, Celltrion, Pfizer, and Galmed. J-FC has received research grants from AbbVie, Pfizer, and Takeda; payment for lectures from AbbVie, Amgen, Pfizer, and Takeda; consulting fees from AbbVie, Amgen, Arena Pharmaceuticals, Boehringer Ingelheim, BMS, Celgene Corporation, Eli Lilly, Ferring Pharmaceuticals, Galmed Research, Genentech, GlaxoSmithKline, Janssen Pharmaceuticals, Kaleido Biosciences, Imedex, Immunic, Iterative Scopes, Merck, Novartis, Otsuka, PBM Capital, Pfizer, Protagonist Therapeutics, Sanofi, Takeda, TiGenix, and Vifor; and holds stock options in Intestinal Biotech Development. EH has received lecture fees from Takeda, Janssen, and BMS; and consultant or advisory board fees from AbbVie, Gilead, and Janssen. All other authors declare no competing interests.
