# Supplementary material for: Withdrawal of infliximab or concomitant immunosuppressant therapy in patients with Crohn's disease on combination therapy (SPARE): a multicentre, open-label, randomised controlled trial
Source: Lancet Gastroenterol Hepatol. 2023 Jan 11;8(3):215–27. doi: 10.1016/S2468-1253(22)00385-5 (PMC9908559; doi:10.1016/S2468-1253(22)00385-5)
Supplement: Supplementary appendix [file mmc1.pdf]

THE LANCET
Gastroenterology & Hepatology

Supplementary appendix

This appendix formed part of the original submission and has been peer reviewed. We post it as supplied by the authors.

Supplement to: Louis E, Resche-Rigon M, Laharie D, et al. Withdrawal of infliximab or concomitant immunosuppressant therapy in patients with Crohn's disease on combination therapy (SPARE): a multicentre, open-label, randomised controlled trial. *Lancet Gastroenterol Hepatol* 2023; published online Jan 11. https://doi.org/10.1016/S2468-1253(22)00385-5.

Appendix

Methods

Important changes to methods after trial commencement (such as eligibility criteria), with reasons

Changes occurred in the trial design during the recruitment period. A first set of changes was designed to improve recruitment. This modification included reduction in the pre-existing duration of combination therapy from 12 months to 8 months; a reduction in duration of stable anti-metabolite dose from 6 months to 3 months; a reduction of stable infliximab dose from 6 months to 4 months; the inclusion of patients on an azathioprine dose lower than 2 mg/Kg or a mercapturine dose lower than 1 mg/Kg if 6TGN level was between 235 and 450 pmol/8.10^8 red blood cells; the inclusion of patients under infliximab biosimilars (without change of biosimilar during the trial); and the introduction of screening procedures within the two months instead of the three weeks preceeding baseline.

Other changes aimed at clarifying some inclusion criteria, including the stratification which was initially according to the duration before the start of infliximab (≤, or > 2 years), changed for the duration before the start of the first TNF (≤, or > 2 years). The list of changes with dates and number of recruited patients is showed in supplementary table 1.

Finally, a recalculation of the sample size was made in November 2018 and explained in the main manuscript.

The successive versions of the study protocol and the number of patients recruited during each period is presented in supplementary table 1.

Results

Secondary endpoints

Factors associated with time to relapse in univariate analysis were: arm B (compared to arm A: HR 3·45 (95% CI: 1·56-7·69) p=0.002; compared to arm C: HR 4·76 (95% CI: 1·92-11·11) p=0.0007), age at diagnosis < 17 yrs (HR 2·87 (95% CI: 1·49-5·53); p=0.002), hsCRP at baseline as a continuous variable (HR 1·11 (95% CI: 1·05-1·18; p=0·0005), ulcers at endoscopy at baseline (HR 2·71 (95% CI: 1·24-5·89; p=0·01), CDEIS at baseline as a continuous variable (HR 1·18 (95% CI: 1·05-1·32); p=0·004), SES-CD at baseline as a continuous variable (HR 1·12 (95% CI: 1·01-1·25); p=0·03), faecal calprotectin > 300 µg/g at baseline (HR 3·16 (95% CI: 1·5-6·65); p=0·002).

In patients stopping infliximab (arm B) factors associated in univariate analysis with the relapse were: total white cell count at inclusion >$6x10^9$/l (HR 2·2 (95% CI: 1-4·83); p=0·049), 6-TGN at baseline>300 pmol/$8x10^8$ RBC (HR 0·23 (95% CI: 0·07-0·69); p=0·009), CDEIS at baseline (HR 1·26 (95% CI: 1·03-1·56); p= 0·028).

Factors associated with treatment failure in univariate analysis were: disease duration (HR 1·04 (95% CI: 1·01-1·08); p=0·015), active smoking compared to never (HR 2·77 (95% CI 1·15-6·67); p=0·02), Montreal B2 stricturing behaviour (HR 3·78 (95% CI: 1·67-8·56); p=0·001), clinically significant stricture at the time of infliximab induction or during infliximab treatment (HR 4·00 (95% CI 1·67-9·58); p=0·002), hsCRP at baseline as continuous variable (HR 1·15 (95% CI 1·09-1·22); p<0·0001), CDEIS at baseline as continuous variable (HR 1·17 (95% CI: 1·02-1·35); p=0·03).

Supplementary table 1

Protocol version	Dates	Nb randomisations
INITIAL	24/07/2014	
AMENDMENT 1 v2	25/06/2015	
AMENDMENT 1 v2.1	24/09/2015	

		26
AMENDMENT 2 V3	27/06/2016	
		91
AMENDMENT 3 V4	19/10/2017	
		23
AMENDMENT 4 V5	15/02/2018	
		71
AMENDMENT 5 V6	03/06/2019	
AMENDMENT 6 V7	31/05/2021	
		211

List of study protocol amendments, with dates and number of included patients.

Supplementary table 2

		**Arm A (n=67)**	**Arm B (n=71)**	**Arm C (n=69)**	**A vs B**	**C vs B**
					**p-value**	**p-value**
hsCRP (mg/l) median [IQR]	Baseline	1·24 [0·52;2·75]	1·15 [0·565;2·205]	1·12 [0·51;2·6]		
	EOS	1·44 [0·67;4]	1·58 [0.685;4]	2 [0·84;5·7]		
	Δ EOS-baseline	0·23 [-0·335;1·875]	0·4 [-0·435;1·75]	0·54 [-0·22;2·35]	0·97	0·72
Faecal calprotectin (µg/g) median [IQR]	Baseline	65·4 [22;311·2]	89 [27;309·5]	78·8 [22;200·9]		
	EOS	73·4 [23·3;306·4]	73·4 [22;263·8]	130·9 [31·2;355·2]		
	Δ EOS-baseline	5·1 [-51·45;121·2]	0 [-66·2;155·2]	39·1 [-16·9;166]	0·38	0·75
CDEIS median [IQR]	Baseline	0 [0;0]	0 [0;0]	0 [0;0]		
	EOS	0 [0;0]	0 [0;2·56]	0 [0;0]		
	Δ EOS-baseline	0 [0;0]	0 [0;0·875]	0 [0;0]	0·28	0·72
SES-CD median [IQR]	Baseline	0 [0;2]	0 [0;0·5]	0 [0;1]		
	EOS	0 [0;1]	0 [0;3·5]	0 [0;1]		
	Δ EOS-baseline	0 [0;1]	0 [0;1·5]	0 [0;1]	0·66	0·096
Infliximab trough (µg/ml) median [IQR]	Baseline	3·6 [2·55;5·6]	4·2 [2·55;6·3]	4·1 [2·5;6]		
	EOS	3·57 [2·35;5·6]	0·08 [0·04;3·57]	3·3 [1;5·8]		
	Δ EOS-baseline	0·2 [-1·05;1·8]	-2·88 [-5·215;-0·525]	-0·75 [-2·2;1·2]	0·021	0·043
Anti-infliximab antibodies (µg/ml) median [IQR]	Baseline	0·4 [0·3;0·6]	0·5 [0·3;0·7]	0·5 [0·3;0·7]		
	EOS	0·4 [0·25;0·6]	0·4 [0·2;0·65]	0·5 [0·3;0·8]		
	Δ EOS-baseline	-0·1 [-0·105;0·1]	-0·1 [-0·2;0·1]	0 [-0·1;0·2]	0·29	0·26

Evolution of endoscopic scores of activity, biomarkers and drug levels between baseline and the end of the study (EOS). Only one imputation dataset was considered for median and IQR to be consistent with Table 2 presented in main manuscript. P.values were computed using Rubin's rules.

Members of the SPARE Biocycle research group collaborative authorship

A Andrews, M Sparrow, R Leong, S Connor, G Radforth-Smith, P De Cruz, F Baert, E Louis, P Bossuyt, M Resche-Rignon, N Ding, S Almer, S Ben-Horin, JF Colombel,B Siegmund, J Preiss, A Stallmach, T Liceni, O Grip, J Halfvarson, D Durai, F Cummings, C Seilinger, M Parkes, J Lindsay, G Lambrecht, P VanHootegem, JF Rahier, M Dewitte, X Hebuterne, E Chanteloup, R Altwegg, S Nancey, G Bouguen, G Pineton de Chambrun, F Pollenot, Y Bouhnik, L Vuitton, M Nachury, D Laharie, S Nancey, M Fumery, G Bouguen, L Picon, C Gilletta, , S Viennot, X Roblin, J Satsangi, J Lindsay, M Parkes, P Irving, C Lamb, D Durai, R Pollok, G D'haens, E Hertervig, O Grip, J Halfvarsson.

List of hospitals having recruited at least one patient in the SPARE trial

**Participating hospitals having randomized patients**	**Number of randomized patients**
St Vincent's hospital, Melbourne, Australia	4
The Alfred hospital, Prahran, Australia	1
Concord Repatriation General Hospital, Sydney, Australia	1
Liverpool Hospital, Australia	1
Royal Brisbane & Women's Hospital, Australia	2
Austin Hospital, Australia	1
Heilig Hartziekenhuis AZ delta, Belgium	5
CHU Liège, Belgium	7
Imelda ziekenhuis, Belgium	8
Clinique Universitaire Saint-Luc, Brussels, Belgium	1
Hôpital Erasme, Brussels, Belgium	1
UZ Gent, Belgium	3
AZ Damiaan ziekenhuis, Oostende, Belgium	3
CHU Namur, Belgium	4
AZ Sint-Lucas, Brugge, Belgium	2
Universitätsmedizin Berlin – Charité, Germany	2
Universitätsklinikum Jena Klinik für Innere Medizin IV , Germany	1
Gastroenterologie am Bayerischen Platz – Berlin, Germany	1
Hôpital St Louis, Paris, France	3
CHU Nantes, France	2
Hôpital Beaujon, Paris, France	6
CHU Reims, France	1
CHU Besançon, France	7
CHU Lille, France	5
CHU Bordeaux, France	20
CHU Lyon, France	3
CHU Amiens, France	5
Hôpital St Antoine, Paris, France	2
CHU Rennes, France	4
CHU Tours, France	9
CHU Toulouse, France	5
CHU Montpellier, France	4
CHU Nice, France	3
CHU Clermont-Ferrand, France	3
CHU Nancy, France	2

CHU Caen, France	6
Hôpital Bicêtre, Paris, France	2
Institut Mutualiste Montsouris, Paris, France	2
CHU St Etienne, France	3
Hôpital St Joseph, Paris, France	5
CH Bayonne, France	1
NHS Lothian, Edinburgh, UK	4
Royal Devon & Exeter NHS Foundation Trust, Exeter, UK	4
Leeds Teaching Hospitals NHS Trust, Leeds, UK	1
University Hospital Southampton NHS Foundation Trust, Southampton, UK	4
Sheffield Teaching Hospitals NHS Foundation Trust, UK	3
Guy's and St Thomas' NHS Foundation Trust, London, UK	7
Newcastle upon Tyne Hospitals NHS Foundation Trust, Newcastle, UK	6
Cardiff and Vale University Health Board, Cardiff, UK	2
London North West University Healthcare NHS Trust, London UK	1
NHS Forth Valley, Falkirk, UK	2
North Tees and Hartlepool NHS Foundation Trust, Stockton-on-Tees, UK	1
Hull and East Yorkshire Hospitals NHS Trust, Hull, UK	2
St George's University Hospitals NHS Foundation Trust, London, UK	6
Hampshire Hospitals NHS Foundation Trust, Winchester, UK	3
Academisch Medisch Centrum, Amsterdam, The Netherlands	3
Skåne university hospital – Lund, Sweden	5
Skåne University hospital, gastroenterology Malmö, Sweden	3
Kliniken Universitetssjukhuset Örebro, Sweden	2
Danderyds Hospital, Sweden	1
**Total of randomized patients**	**211**
